# Mitophagy in Pancreatic Cancer

**DOI:** 10.3389/fonc.2021.616079

**Published:** 2021-02-26

**Authors:** Yangchun Xie, Jiao Liu, Rui Kang, Daolin Tang

**Affiliations:** ^1^ Department of Oncology, The Second Xiangya Hospital, Central South University, Changsha, China; ^2^ The Third Affiliated Hospital, Guangzhou Medical University, Guangzhou, China; ^3^ Department of Surgery, UT Southwestern Medical Center, Dallas, TX, United States

**Keywords:** mitophagy, autophagy, PDAC - pancreatic ductal adenocarcinoma, tumorigenesis, therapy

## Abstract

Pancreatic ductal adenocarcinoma (PDAC), one of the most aggressive solid malignancies, is characterized by the presence of oncogenic KRAS mutations, poor response to current therapies, prone to metastasis, and a low 5-year overall survival rate. Macroautophagy (herein referred to as autophagy) is a lysosome-dependent degradation system that forms a series of dynamic membrane structures to engulf, degrade, and recycle various cargoes, such as unused proteins, damaged organelles, and invading pathogens. Autophagy is usually upregulated in established cancers, but it plays a dual role in the regulation of the initiation and progression of PDAC. As a type of selective autophagy, mitophagy is a mitochondrial quality control mechanism that uses ubiquitin-dependent (e.g., the PINK1-PRKN pathway) and -independent (e.g., BNIP3L/NIX, FUNDC1, and BNIP3) pathways to regulate mitochondrial turnover and participate in the modulation of metabolism and cell death. Genetically engineered mouse models indicate that the loss of PINK1 or PRKN promotes, whereas the depletion of BNIP3L inhibits oncogenic KRAS-driven pancreatic tumorigenesis. Mitophagy also play a dual role in the regulation of the anticancer activity of certain cytotoxic agents (e.g., rocaglamide A, dichloroacetate, fisetin, and P. suffruticosa extracts) in PDAC cells or xenograft models. In this min-review, we summarize the latest advances in understanding the complex role of mitophagy in the occurrence and treatment of PDAC.

## Introduction

More than 90% of pancreatic cancers are ductal adenocarcinoma (PDAC), which is highly malignant, difficult to diagnose early, and has a very poor prognosis. It is estimated that by 2030, pancreatic cancer will become the second largest tumor-related death in humans (1). Although there have been a variety of “precision” targeted therapies for certain solid cancers (such as lung and breast cancer), the clinical treatment of PDAC is still in the “non-precision” era. The effective rate of the widely used gemcitabine regimen is only 30%, while FOLFIRINOX (a regimen consisting of 5-fluorouracil, leucovorin, irinotecan, and oxaliplatin) has serious adverse reactions, and the targeted drug erlotinib (an oral tyrosine kinase inhibitor of epidermal growth factor receptor [EGFR]) as well as cutting-edge immune checkpoint inhibitors have limited efficacy in patients with PDAC (2). How to achieve “precise” diagnosis and treatment of PDAC is a challenging issue in clinical practice. This clinical goal may require in-depth basic research to understand the complex pathological mechanisms of PDAC initiation and development.

Cells produce a large amount of waste every day, which needs to be removed by an integrated degradation system to maintain normal cell functions. In addition to the ubiquitin-proteasome system (UPS), autophagy is a lysosomal-dependent pathway that can remove various endogenous cellular materials (such as proteins and organelles) and exogenous invading pathogens. Autophagy dysfunction (including defects or over-activation) may cause abnormal cell components and functions, leading to various pathological conditions and diseases (3). Therefore, it is important to understand the types, functions, and regulation of autophagy under different conditions (4). The focus on autophagy provides a promising alternative to the development of new treatment options for human diseases. In this min-review, we describe the types of autophagy and the mechanism of mitophagy, and then analyze the effects of mitophagy on PDAC, including tumorigenesis and tumor treatment.

## Type of Autophagy

**Figure 1:**
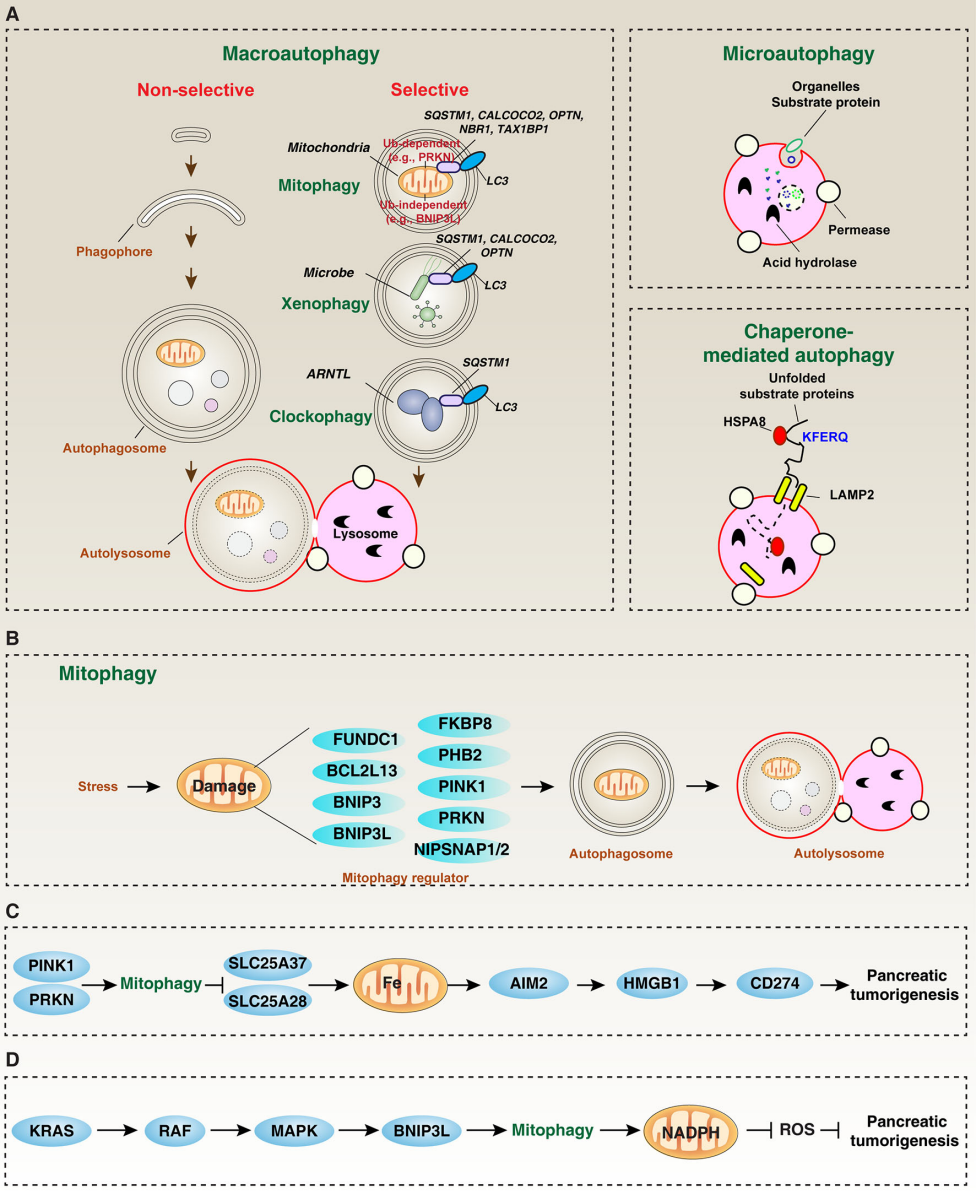
The role of mitophagy in pancreatic tumorigenesis. **(A)** In mammalian cells, there are three main types of autophagy: microautophagy, macroautophagy, and chaperone-mediated autophagy. Macroautophagy can be further divided into selective and non-selective forms. **(B)** Core mitophagy regulators mediate mitochondrial clearance. **(C, D)** PINK1/PRKN and BNIP3L-dependent mitophagy play different roles in inhibiting or promoting pancreatic tumorigenesis, respectively.

According to the different ways of transporting cellular components to lysosomes, autophagy is divided into the following categories (**Figure 1A**) (4). 1) Macroautophagy. The process of macroautophagy is a dynamic membrane reforming process involving the formation and maturation of three special structures: phagophore (also known as separated membrane produced by endoplasmic reticulum, mitochondria, or other subcellular membrane organelles), autophagosome (a double-membrane organelle phagocytosing degradable materials), and autolysosome (a hybrid organelle formed by the fusion of autophagosomes and lysosomes) where sequestered material is degraded by lysosomal hydrolases. 2) Microautophagy: lysosome membrane directly envelops longevity protein and then degrades in lysosome; 3) Chaperone-mediated autophagy (5): proteins containing KFERQ-like motifs bind to molecular chaperones (such as heat shock protein family A (Hsp70) member 8 [HSPA8/HSC70]), and then are transported to the lysosome cavity by lysosomal associated membrane protein 2 (LAMP2/LAMP2A) to be digested by lysosomal enzymes. It is worth noting that the multimerization of LAMP2 is required to transport the substrate into the lysosomal cavity (6, 7). Among them, macroautophagy (hereinafter referred to as autophagy) is the most common and well-studied form of autophagy in mammalian cells. The so-called autophagy-related (ATG) genes or proteins play a key role in the regulation of autophagy membrane dynamics through protein-protein interaction, and post-translational modifications (especially phosphorylation) further regulate autophagic process by affecting ATG function (8).

According to the selectivity of the substrate to be degraded, autophagy is further divided into selective autophagy and non-selective autophagy to control cell fate (9, 10) (**Figure 1A**). Non-selective autophagy refers to non-specific degradation processes, such as starvation-induced autophagic degradation. In addition to the core autophagy mechanism, selective autophagy also requires specific autophagy receptors to selectively degrade specific cargo (9, 11, 12). For example, xenophagy (13), clockophagy (14, 15), and mitophagy (16) can selectively degrade invading pathogens, aggregated circadian protein aryl hydrocarbon receptor nuclear translocator like (ARNTL), and damaged mitochondria, respectively (**Figure 1A**). This selective autophagy mainly depends on the molecular bridge-like autophagy receptor (also called adaptor protein), which not only specifically binds to the substrate, but also binds to members of the ATG8/LC3 family (MAP1LC3A, MAP1LC3B, MAP1LC3C, GABARAP, GABARAPL1/GEC1, GABARAPL2/GATE-16, and GABARAPL3) through different structure domains (12). The number of genes in the ATG8/LC3 family may be caused by gene duplication and loss events during evolution. LC3-II is a standard marker for autophagosomes, which is produced by conjugating cytoplasmic LC3-I with phosphatidylethanolamine on the surface of newborn autophagosomes (17). It is worth noting that certain autophagy receptors (such as sequestosome 1 [SQSTM1/p62]) act on both selective and non-selective autophagy during stress (18). In addition, the protein level of SQSTM1 is also regulated by the crosstalk between the UPS and autophagy pathways (19). Collectively, these kinetics indicate that a complex feedback network is involved in metabolism and signal transduction to control substrate degradation (20).

## Type of Mitophagy

Mitochondria are organelles composed of two membranes (inner membrane and outer membrane) found in most cells. Normal mitochondria act as a “power factory” whose main function is to perform aerobic respiration to produce adenosine triphosphate (ATP). In addition, the interaction between mitochondrial and non-mitochondrial metabolic pathways is important for generating secondary signals (such as reactive oxygen species [ROS] and calcium) and biological macromolecules (such as proteins, carbohydrates, lipids, and nucleic acids). Therefore, maintaining healthy mitochondria, including quantity and quality, is essential for cell homeostasis. Conversely, damage to the mitophagy pathway can cause various pathological conditions (such as inflammation) and diseases (such as neurodegenerative diseases and cancer) (21–23). As an important component of the mitochondrial quality control mechanism, mitophagy can be activated through either ubiquitin (Ub)-dependent or independent pathway (**Figure 1A**), which is regulated by various proteins, including mitochondrial inner or outer membrane proteins (**Figure 1B**).

Mitophagy that rely on Ub can be further divided into classical and non-classical pathways (24). The classical pathway is mediated by the PTEN induced kinase 1 (PINK1, a serine–threonine protein kinase) and parkin RBR E3 ubiquitin protein ligase (PRKN/PARK2) (25). Mutations in PINK1 and PRKN are one of the important causes of Parkinson’s disease, a progressive neurodegenerative disease with motor and non-motor symptoms. The impaired PINK1-PRKN-dependent mitophagy pathway also promotes various types of tumor formation, including PDAC (discussed later). Mechanistically, oxidative damage to the mitochondria causes the accumulation of PINK1 on the mitochondrial outer membrane and the recruitment of PRKN from the cytoplasm to the mitochondria, leading to subsequent assembly of phosphorylated Ub chains on mitochondrial outer membrane proteins (25). In addition to the earliest reported SQSTM1 (25), other autophagy receptors, such as optineurin (OPTN) (26), neighbor of BRCA1 gene 1 (NBR1) (27), calcium binding and coiled-coil domain 2 (CALCOCO2/NDP52) (28), and tax1 binding protein 1 (TAX1BP1) (28), also help to recognize and degrade damaged mitochondria after activating the PINK1-PRKN pathway (**Figure 1A**). Moreover, PINK1-PRKN-mediated mitophagy can be reversed by deubiquitinating enzymes, such as ubiquitin-specific peptidase 8 (USP8), USP15, USP30, and USP35 (29). Non-classical Ub-dependent mitophagy is mediated by non-PRKN E3 ubiquitin ligases (such as mitochondrial E3 ubiquitin protein ligase 1 [MUL1] (30), siah E3 ubiquitin protein ligase 1 [SIAH1] (31), SMAD specific E3 ubiquitin protein ligase 1 [SMURF1] (32), and autocrine motility factor receptor [AMFR/GP78]) (33). The impact of crosstalk between Ub-dependent classical and non-classical mitophagy pathways on tumors is still poorly understood.

Ub-independent mitophagy is mediated by receptors, rather than E3 ligases. Recently, depending on the stimulus and cell type, the list of mitophagy receptors is increasing (34). In addition to the early reports of BCL2 interacting protein 3 like (BNIP3L/NIX) acting as a mitophagy receptor in red cells (35), other mitophagy receptors, including FUN14 domain containing 1 (FUNDC1) (36), BCL2 interacting protein 3 (BNIP3) (37), nipsnap homolog 1 (NIPSNAP1) (38), nipsnap homolog 2 (NIPSNAP2) (38), prohibitin 2 (PHB2) (39), BCL2 like 13 (BCL2L13) (40) and FKBP prolyl isomerase 8 (FKBP8) (41), have also been identified in cancer and non-cancer cells (**Figure 1B**). These unique receptors are responsible for binding to different mitochondrial membrane components in response to various stresses (such as hypoxia and oxidative damage). In addition to protein, non-protein mitochondrial components, such as cardiolipin and ceramide (42), also mediate mitophagy in some case, indicating that there are complex mitophagy sub-routes to regulate mitochondrial turnover and function.

## Mitophagy in Pancreatic Cancer

Compared to normal cells, pancreatic cancer cells generally exhibit highly fragmented mitochondria, which is associated with increased mitochondrial fission and numbers as well as enhanced mitochondrial oxidative phosphorylation or glycolysis (43–45). Therefore, understanding the mechanism of mitochondrial biogenesis and turnover in different stages of pancreatic cancer, including initiation, progression, and metastasis, is essential for the next generation of cancer treatments. Indeed, increased autophagy or mitophagy levels are observed in various types of pancreatic cancer (46–48). However, autophagy plays a dual role in various cancer (including PDAC), depending on many factors, such as tumor stage, tumor microenvironment, gene mutation status involving oncogenes and tumor suppressor genes, and metabolic reprogramming (49–54). PDAC is a heterogeneous disease and can be morphologically classified into four types: conventional, tubulo-papillary, squamous, and “composite”, which exhibit different molecular and genetic characteristics (55). Generally, autophagy inhibits the growth of PDAC in the early stage by limiting DNA damage or inflammation, and upregulated autophagy in the later stage can promote PDAC survival by limiting cell death or anti-tumor immunity (56–60). Since covering all the effects of autophagy in PDAC is outside the scope of this min-review, we will only discuss the modulation and function of mitophagy in PDAC as described below.

### Pancreatic Tumorigenesis

Evidence is accumulating that both intrinsic genetic factor and extrinsic environmental factor are important for tumorigenesis. For pancreatic cancer, the oncogenic KRAS mutation is a key driving force for the formation of precursor lesions and subsequent development of PDAC with stromal response (61). KRAS activation is related to changes in mitochondrial morphology (e.g., increased mitochondrial fragmentation) and function (for example, reduction of mitochondrial respiratory complex I activity, enhancement of glycolytic activity, promotion of ROS production and induction of mitophagy) in various cancers (including PDAC) (62–66). Moreover, the conditional expression of endogenous *Kras^G12D^* in the pancreas of mice (*Pdx1-Cre;Kras^G12D^*; called KC mice) can mimic most of pathological development of human PDAC (67). This spontaneous transgenic PDAC mouse model is widely used to further consume or overexpress additional genes to evaluate the function of target genes in pancreatic tumorigenesis (**Table 1**). For example, based on KC mice, further depletion of the tumor suppressor high mobility group box 1 (HMGB1, *Pdx1-Cre;Kras^G12D^;Hmgb1^-/-^*) (69) or overexpression of tumor protein p53 (TP53) mutation (*Pdx1-Cre;Kras^G12D^;Tp53^R172H^*, termed KPC mice) (70) can significantly promote the development of KRAS-driven PDAC. HMGB1 is a positive regulator of autophagy and mitophagy, coupled with TP53 signaling in a variety of tumors (71–74). Cytoplasmic HMGB1 is a BECN1-binding protein that contributes to the formation of autophagosomes (72). Nuclear HMGB1 promotes the expression of heat shock protein β-1 (HSPB1) and subsequent HSPB1-mediated cytoskeletal integrity, which is required for the membrane dynamics of mitophagy (71). In addition, mitochondrial HMGB1 repairs mitochondrial genomic DNA damage, which also plays a potential role in suppressing tumorigenesis (75). However, depletion of mitophagy regulators, including PINK1 [*Pdx1-Cre;Kras^G12D^;Pink1^-/-^*] (22), PRKN [*Pdx1-Cre;Kras^G12D^;Prkn^-/-^*] (22), or BNIP3L/NIX [*Pdx1-Cre;Kras^G12D^;Bnip3l^-/-^ or Pdx1-Cre;Kras^G12D^;Tp53^R172H^; Bnip3l^-/-^* (68), in KC mice exhibits different phenotype in pancreatic tumorigenesis. These transgenic animal studies show that Ub-dependent and independent mitophagy pathways play different roles in PDAC.

**Table 1:** Mitophagy regulators in PDAC.

Mitophagy regulator	Expression in human PDAC	Function	Mechanism	Refs
BNIP3L	Upregulation	Tumor promoter	Increases glucose metabolism and antioxidant capacity	(68)
PINK1	Upregulation	Tumor suppressor	Inhibits inflammation and mitochondrial iron-related antitumor immunity	(22)
PRKN	Downregulation	Tumor suppressor	Inhibits inflammation and mitochondrial iron-related antitumor immunity	(22)
HMGB1	Upregulation	Tumor suppressor	Inhibits genomic instability and mitochondrial dysfunction	(69)
TP53	Upregulation	Tumor suppressor	Inhibits genomic instability and mitochondrial dysfunction	(70)

Dysregulated autophagy promotes or inhibits the growth of pancreatic cancer by interfering with different metabolic pathways or tumor signals, such as carbohydrate metabolism, fatty acid β-oxidation, and amino acid transport (48). For example, the reduced glycolysis gene PKM2 promotes survival by maintaining autophagy induced by low glucose in PDAC cells (76). Autophagy-mediated lipid degradation and subsequent fatty acid β-oxidation may provide additional resources for ATP production during PDAC growth (77, 78). Autophagy-mediated degradation of cellular material provides reusable amino acids for PDAC cell proliferation during glutamine deprivation (79). In addition, the PINK1-PRKN pathway can degrade mitochondrial iron importers (such as solute carrier family 25 member 37 [SLC25A37] and solute carrier family 25 member 28 [SLC25A28]) through SQSTM1-mediated mitophagy to inhibit carcinogenic KRAS-driven pancreatic tumorigenesis in mice, thereby inhibiting mitochondrial iron-mediated absent in melanoma 2 (AIM2)-dependent inflammasome activation and the subsequent activation of damage associated molecular pattern (DAMP, such as HMGB1)-dependent immune checkpoint expression (e.g., CD274/PD-L1) (**Figure 1C**) (22). These findings establish a role of PINK1/PRKN-mediated mitophagy to inhibit pancreatic tumorigenesis by limiting chronic inflammation-related immunosuppression in the hypoxic tumor microenvironment (80). Of note, high expression of *PRKN* mRNA was found to be associated with improved survival of pancreatic cancer patients, whereas mRNA expression of *PINK1* did not influence patient survival (22), indicating that PINK1 is a contributor of PDAC, but it is not a potential biomarker. In addition, both PINK1 and PRKN may have mitophagy-independent functions in controlling the quality of mitochondria during pancreatic tumorigenesis (22).

In contrast, in a precursor lesion called pancreatic intraepithelial neoplasia (PanIN), oncogenic KRAS-mediated BNIP3L expression may activate mitophagy in a rapidly accelerated fibrosarcoma (RAF)-mitogen-activated protein kinase (MAPK)-dependent manner to limit the flux of glucose to mitochondria and enhance reduced nicotinamide adenine dinucleotide phosphate (NADPH)-dependent redox capacity, thereby promoting pancreatic tumorigenesis (**Figure 1D**) (68). In KC and KPC pancreatic cancer models, the depletion of additional BNIP3L will increase the content of mitochondria in PanIN, thereby increasing the production of mitochondrial ROS to limit the development of PanIN to PDAC (68). These observations indicate that BNIP3L-mediated mitophagy have different roles in promoting pancreatic tumorigenesis by enhancing the antioxidant capacity of cancer cells for cell proliferation and metastasis. However, oxidative stress and redox regulation are double-edged swords in tumorigenesis (81). Certain types of oxidative cell death, such as necroptosis (a caspase-independent regulated necrosis) and ferroptosis (an iron-dependent regulated necrosis), can promote KRAS-driven PDAC by activating inflammation-related immune suppression (82–84). Whether PINK1, PRKN2, and BNIP3L have non-mitochondrial functions in the modulation of the oncogene KRAS signal remains to be seen. In addition, various types of regulated cell death are closely related to autophagy (85–87), which may accelerate the complexity of the immune characteristics of the tumor microenvironment, thereby affecting anti-tumor immunity.

There is emerging evidence that impaired mitophagy is related to epithelial-mesenchymal transition and pancreatic cancer stem cells (pCSCs), which are pluripotent, self-renewable, and capable of forming tumors (88). In particular, the interferon signaling pathway-mediated the upregulation of Ub-like modifier interferon-stimulated gene 15 (ISG15) and its modification ISGylation maintain mitophagy and metabolic plasticity of pCSCs (89), suggesting a potential link between interferon, mitophagy, and metabolism in pCSCs. PDAC patients with high ISG15 levels showed increased expression of genes related to the CSC pathway, including epithelial-mesenchymal transition and oxidative phosphorylation (89). In contrast, the inhibition of ISG15/ISGylation impairs PRKN-dependent mitophagy, causing pCSCs to fail to eliminate dysfunctional and unhealthy mitochondria (89). Overall, these findings support a role of ISG15 in pCSCs by regulating mitochondrial dynamics and energy metabolism. The role of ISG15 in pancreatic tumorigenesis needs to be further studied using transgenic mice.

### Pancreatic Cancer Therapy

The purpose of tumor treatment is to induce death in tumor cells without damaging normal cells. Cell death can be divided into accidental or regulated cell death (90). Regulated cell death further includes apoptotic and non-apoptotic forms. In addition to the extensively studied apoptosis (91, 92), the induction of non-apoptotic regulated cell death [such as necroptosis (93, 94), alkaliptosis (95, 96), and ferroptosis (97–101)] in preclinical PDAC models has shown promising results in inhibiting tumor growth. As a metabolic center, mitochondria play a complex role in regulating apoptosis and non-apoptotic cell death by cooperating with other subcellular organelles (102). Accordingly, mitophagy-mediated mitochondrial degradation and turnover is reasonable to affect the anti-cancer activity of cytotoxic agents in PDAC cells. The best cell models for studying mitochondrial biology and mitophagy of pancreatic cancer are various human PDAC cell lines with KRAS mutations. For example, rocaglamide A, a natural product from the plant Aglaia elliptifolia, has the ability to induce PINK1/PRKN-mediated mitophagy as a negative feedback mechanism to limit rocaglamide A-induced apoptosis in various human PDAC cell lines with KRAS mutations (103). In contrast, the inhibition of mitophagy by Mdivi-1 enhances the anti-cancer activity of rocaglamide A in PDAC cells (103). Overexpression of serine/threonine kinase 25 (STK25, also known as MST1) in various PDAC cells induces apoptosis by inhibiting mitophagy mediated by mitofusin 2 (MFN2) (104). In contrast, leflunomide, an FDA-approved arthritis drug, can inhibit the growth of PDAC tumors by inducing MFN2 expression and subsequent mitophagy (44). In addition, *in vitro* and xenograft models, the combination of cyst(e)inase (an engineered human enzyme) and anuranofin (a thioredoxin reductase inhibitor) can inhibit mitophagy, thereby increase ROS production and apoptosis in the human PDAC cells (105). In other cases, dichloroacetate (an inhibitor of pyruvate dehydrogenase kinase) (106), fisetin (a bioactive flavonoid molecule found in fruits and vegetables) (107), and P. suffruticosa extracts (108) may play a context-related role in the induction of mitophagy and tumor suppression in PDAC cells. These findings further indicate that the complex relationship between mitophagy and mitochondrial dynamics can affect the effects of chemotherapy and targeted therapy.

In addition to PDAC cells, pCSCs is another cell model for studying mitochondrial dysfunction. pCSCs not only promote the growth and metastasis of pancreatic tumors, but also mediate chemoresistance. Metformin is a biguanide anti-diabetic drug that activates AMP-activated protein kinase (AMPK) to trigger autophagy (109). Retrospective studies have shown that compared with patients receiving insulin or sulfonylureas, many diabetic patients with solid tumors (including pancreatic cancer) treated with metformin have a survival benefit (109). The loss of ISG15 in pCSCs by CRISPR-Cas9 technology results in sensitivity to metformin therapy in xenograft models (89). These findings further indicate a potential role of ISG15 in regulating the anti-cancer activity of metformin in pCSCs. Further investigations are still needed to determine whether ISG15 directly regulates AMPK activation in pCSCs.

## Conclusion and Perspectives

In the past decade, basic and clinical research on autophagy has involved various diseases, including pancreatic cancer (48, 59, 110–112). With the deepening of research, the functions of autophagy in tumor biology show diversity and complexity. One of the important reasons is that autophagy can have different degradation substrates, and these substrates can play a tumor-promoting and anti-tumor effect. In addition, the degree of substrate degradation (such as complete or partial degradation) also affects the function of autophagy in tumors. Similarly, mitochondrial coupling with mitochondrial biogenesis also plays a dual role in cancer. In this min-review, we discussed the context-dependent role of mitophagy in pancreatic cancer. Although this information enhances our understanding of the role of mitochondrial homeostasis in pancreatic cancer, there are still some key questions about the process and function of mitophagy in PDAC. How does the multi-step mitophagy actually proceed at different stages of PDAC? What are the key molecules or signals that distinguish the functions of mitophagy in promoting or inhibiting pancreatic tumorigenesis? Do tumor cells and non-tumor cells (such as immune cells or stromal cells) in the pancreatic tumor microenvironment have different mitophagy activities? In the pancreatic tumor microenvironment, what is the synergy or competition between mitophagy and other types of selective autophagy? How to develop specific mitophagy targeted drugs to kill pancreatic tumors? Are there specific markers to assess the level of mitophagy in PDAC patients?

## Author Contributions

YX and DT conceived of the topic for this review. All authors contributed to the article and approved the submitted version.

## Funding

YX was supported by the National Natural Science Foundation of China (No. 81802476).

## Conflict of Interest

The authors declare that the research was conducted in the absence of any commercial or financial relationships that could be construed as a potential conflict of interest.
